# Effects of backhand stroke styles on bone mineral content and density in postmenopausal recreational tennis players: a cross-sectional pilot investigation

**DOI:** 10.1186/s12905-021-01416-z

**Published:** 2021-07-29

**Authors:** Ho-Seng Wang, Yi-Shan Tsai, Yung-Chih Chen, Hsiao-Han Chao, Hsin-Shih Lin, Yi-Pin Chiang, Hou-Yu Chen

**Affiliations:** 1 Department of Physical Education and Sport Sciences, National Taiwan Normal University, No. 162, Section 1, Heping East Road, Taipei City, 106 Taiwan; 2 Department of Athletics, National Taiwan University, No. 1, Sec. 4, Roosevelt Road, Taipei City, 10617 Taiwan; 3 Institute of Physical Education, Health and Leisure Studies, National Cheng Kung University, No. 1, University Road, Tainan City, 701 Taiwan; 4 Department of Rehabilitation Medicine, Mackay Memorial Hospital, No. 92, Sec. 2, Zhongshan N. Road, Taipei City, 10449 Taiwan; 5 Education Center for Humanities and Social Sciences (ECHSS), National Yang Ming Chiao Tung University, No. 155, Sec. 2, Linong Street, Taipei City, 112 Taiwan

**Keywords:** Tennis, Osteoporosis, Fracture, Lumber, Bone mineral density

## Abstract

**Background:**

One-handed backhand (OB) and two-handed backhand (TB) styles are commonly used in tennis, but only TB generates loadings on the non-dominant arm and a greater extension torque on the rear leg, leading to a greater axial torque involving rotation of the hip and trunk. The current study investigated whether those effects can further affect bone area (BA), bone mineral content (BMC) and density (BMD) in postmenopausal recreational tennis players.

**Methods:**

BA, BMC and BMD of the lumbar spine, hip and distal radius were assessed using dual-energy X-ray absorptiometry in TB, OB, and swimmers’ group as a control (SG) (all participants self-reported for at least 5 years of exercise history, n = 14 per group). Muscular strength was assessed with a hand dynamometer. Among these three groups, the BA, BMC and BMD of distal radius and muscle strength were assessed using one-way ANOVA, and those of the lumbar region and the hip joint were tested by one-way ANCOVA.

**Results:**

TB showed higher BMC and BMD for both lumbar spine and femoral neck than SG (all, *p* < 0.05). Both OB and TB showed greater BMD inter-trochanter than SG (both, *p* < 0.05). OB demonstrated greater inter-arm differences in the distal radius, which involved 1/3 distal for BMC and mid-distal radius for BMD compared to the TB and SG (all, *p* < 0.05). In addition, greater inter-arm asymmetry of grip strength was found in OB compared to TB and SG (both, *p* < 0.05).

**Conclusion:**

For postmenopausal women, performing two-handed backhand strokes, leads to higher BMC and BMD in the non-dominant arm, the lumbar region, and hips, indicating potential benefit to maintain bone health and strength. Whether this result leads to reducing the risk of osteoporosis needs to be investigated in further research.

## Introduction

There are millions of fracture cases annually worldwide due to low bone mass/density and/or osteoporosis. In women, bone loss, namely a reduction in bone mass and/or bone density, accelerates during the menopause, especially during the 2 years before and the 4–5 years after the last menses [1]. This means that postmenopausal women face a higher incidence of osteoporosis and a greater risk of bone fracture compared to men [1]. In addition, among women over 45 years old, osteoporosis has a longer effect than other diseases such as diabetes, heart attack or breast cancer [2]. Overall, women with postmenopausal in particular are highly susceptible to the devastating effects of osteoporosis and fractures.

Calcium supplementation has been proven effective in maintaining bone mineral content (BMC) and bone mineral density (BMD) for postmenopausal women, but the potential adverse effects argue for other complementary treatments/interventions [3]. Studies have demonstrated that impact exercise and/or weight-bearing physical activity are able to effectively attenuate bone loss in the lumbar spine and femoral neck areas among postmenopausal women [4, 5]. Tennis is both a weight-bearing and an impact form of exercise and has been recommended for maintaining BMC and BMD [4]. Moreover, tennis is a (mostly) unilateral sport for the upper extremity and that the trunk and lower extremities are loaded. It has been shown to result in a higher BMC and BMD value for the dominant arm that carries out forehand strokes [6–8]. Thus tennis exercise results in significant inter-arm asymmetry among postmenopausal amateur tennis players [6], which suggests that such exercise is having positive effects in terms of impact loadings on bone structure over time [6].

In addition to the forehand stroke, the backhand stroke is also one of the most heavily used basic techniques in tennis. This stroke can be performed in two different styles; these are one-handed and two-handed. During one-handed backhand stroke, the swing mainly relies on the dominant arm, thus high mechanical loadings and greater impact is observed that affect the dominant arm [9]. By way of contrast, the non-dominant arm plays a much more important role in two-handed backhand strokes (e.g., greater velocities whilst performing the stroke) [10] and this stroke also appears to result in more vibration from the collision with the ball [10]. Studies have also shown the putative role of the muscles in the mechanical loading on bones [9, 11, 12]. Moreover, two-handed backhand strokes performed by young tennis players have demonstrated a lower side-to-side difference in cortical volume [13]. As a result, we hypothesized that two-handed backhand players might have benefited from this stroke because they received more mechanical loading and impact, which should result in an increase in BMC and BMD values for the non-dominant arm. In addition to arm-related specific effects, for the whole body, the two-handed backhand style during the acceleration phase generates greater extension torque in the rear leg (i.e., back leg in the backhand stance) compared to the one-handed backhand style, leading to a larger axial torques during rotation of the hip and trunk [10, 14, 15]. Collectively, it is possible that these two different types of backhand stoke might induce differential mechanical loadings on the hands, arms and trunk, as well as on various lower body bone structures.

Currently, the effects of the different styles of backhand stroke, to the best of our knowledge, have never been investigated among postmenopausal women who are at a higher risk of suffering from osteoporosis and fracture. Therefore, the purpose of this study was to investigate whether performing one-handed or two-handed backhand strokes might have an effect on various bone factors (e.g. BMC and BMD in lumbar spine, hip joints and distal radius) among recreational postmenopausal female tennis players.

## Materials and methods

### Experimental design

Fourteen one-handed backhand (OB) and fourteen two-handed backhand (TB) postmenopausal (at least 6 months after last menses) recreational female tennis players were recruited into the current study via a local advertisement. Both the OB and the TB groups self-reported that they have been playing tennis for at least 5 years using the same backhand technique and that they had been doing this at least twice per week for the last two years. In order to understand whether weight-bearing bilateral/unilateral impact exercise can affect bone responses, another fourteen postmenopausal (also at least 6 months after last menses) recreational female swimmers (SG) were recruited as a control group since swimming has been considered as a non-weight bearing exercise. The SG also self-reported to have been swimming for at least 5 years and that they have been doing this twice per week with more than one hour in each session for the past two years. Moreover, the SG had no unilateral exercise training experience throughout the lifespan. All the participants were healthy (e.g., without diabetes and cardiovascular diseases), right-handed, without any fracture history, and were not using any medications and/or supplements that might affect bone metabolism.

The weekly exercise hours for both the OB and the TB groups were higher than those of the SG group (*p* < 0.05, Table 1) owing to the nature of tennis exercise, which involves intermittent breaks at each point scored, but there was no difference in the weekly exercise hours between the OB and the TB.

**Table 1:** Participants’ characteristics (means ± SD)

	OB (n = 14)	TB (n = 14)	SG (n = 14)
Age (years)	59.2 ± 6.1	58.2 ± 4.4	57.1 ± 5.0
Height (cm)	159.4 ± 7.4	157.5 ± 3.9	157.1 ± 3.5
Total body mass (kg)	60.7 ± 8.6	55.4 ± 5.2	59.8 ± 8.4
Total lean mass (kg)	39.5 ± 5.7	35.5 ± 2.8	37.6 ± 4.9
Total body fat (%)	31.7 ± 5.0	31.8 ± 5.1	33.2 ± 4.7
Menopausal time (years)	8.3 ± 5.9	7.4 ± 5.8	5.7 ± 4.7
Starting age of playing (years)	34.9 ± 8.8	39.1 ± 7.1	39.4 ± 5.3
Sport-specific practice (sessions/week)	4.7 ± 2.1	4.9 ± 1.3	5.6 ± 0.9
Sport-specific practice (hours/week)	8.3 ± 4.1*	7.5 ± 2.5*	3.4 ± 1.2

OB, One-handed backhand; TB, two-handed backhand; SG, swimmers’ group

^*^Greater than SG, *p* < 0.05

The study protocol was approved by TMU-Joint Institutional Review Board (Approval No. 201101015). Written informed consent was obtained from all participating individuals before they were included in the study.

### Bone factors and body composition assessment

Bone factors and body composition were assessed by dual-energy X-ray absorptiometry (DEXA, Delphi QDR Series; Hologic Inc., MA, USA) at the Mackay Memorial Hospital in Taipei, Taiwan. Bone factors consisted of bone area (BA), BMC of the lumbar spine, of a hip joint, of the distal radius and BMD of the lumbar spine, of a hip joint and of a distal radius. These values were used to investigate whether the different backhand stroke styles might influence abovementioned bone responses. The details of the examined regions are described as follows.

The lumbar spinal discs between L2 and L4 were used to represent the lumbar spine [16]. The left hip was defined as the hip joint including the value of femoral neck, greater trochanter, inter-trochanter, and Ward’s triangle [16]. The Ward’s triangle was defined as the area (approximately 1.1 cm^2^) of the femoral neck with the lowest BMD [16]. Distal radius was further divided into ultra-distal (UD), mid-distal (MID) and one-third distal (1/3). The UD region, which consists mainly of trabecular bone, was defined as the region forming a 1.5-cm band next to the end plate of radius, while the 1/3 region, which is comprising mostly of cortical bone, was defined as the 2-cm region 1/3 of the distance between ulnar styloid and olecranon. The MID was defined as the remaining region between the UD and the 1/3 [17]. Asymmetry between one side of the body and the other was expressed as the percentage difference between the two arms and was calculated using the following equation: Δ% = (dominant − nondominant)/nondominant × 100 [13].

### Muscle strength assessment

Muscle strength (including grip strength and isometric wrist flexion strength) was also tested. Grip strength was measured twice for each hand with a 2 min break between reading using a digital hand dynamometer (Grip-D, TKK5401; Takei Scientific Instruments, Niigata, Japan). Participants were instructed to hold the dynamometer while standing with the elbow at a 90° angle and the highest value was recorded [18]. Isometric wrist flexion strength was accessed using a Biodex system 4 Pro (Biodex Medical Systems, NY, USA). Participants sat in a chair with the back at an 85° angle, the wrist in a neutral posture (0°) and the forearm pronated. Maximal voluntary contraction was assessed three times for each hand at intervals of 60 s, and the highest value was recorded [19].

### Statistical analysis

All data are shown as means ± standard deviation (SD). The homogeneity of the parameters was examined by the Levene’s test. One-way ANOVA was used to assess the differences of distal radius (also for the UD, MID and 1/3 distal) among groups (OB, TB and SG) for the BA, BMC and BMD values obtained from the dominant and non-dominant. One-way ANOVA was also carried out for percentage differences of muscle strength. One-way ANCOVA, adjusting for age, height, body mass and menopausal age, was applied to identify the differences among the groups in terms of BA, BMC and BMD values for lumbar region and hip joint (also for the femoral neck, greater trochanter, inter-trochanter, and Ward’s triangle). Bonferroni *post-hoc* comparison was then performed [20]. Effect size (Cohen’s *f* is for ANOVA) were calculated using the formula published by Cohen [21]. The thresholds for Cohen’s *f* for small, moderate and large effects were defined as 0.1, 0.25 and 0.4, respectively. Data were analyzed using the program SPSS 20.0 for Windows. Statistical significance was set at α = 0.05.

## Results

### BA, BMC and BMD of the lumbar spine and of the hip

Both the BMC and BMD values for the TB group measured for the lumbar spine and femoral neck were higher than the SG group values (*p* < 0.05, Table 2). Both the BMC and BMD values from the lumbar spine and femoral neck areas among the OB group were also greater than among the SG groups, although this was not statistically significant. Both the OB and TB groups showed greater BMD values at the inter-trochanter compared to the SG group (*p* < 0.05, Table 2). However, there was no differences in BA values for the lumbar spine and hip joint values across the three groups.

**Table 2:** BA, BMC and BMD at lumbar and hip joint (means ± SD)

	OB	TB	SG	Effect size (*f*)
*BA (cm*^*2*^*)*				
Lumbar (L2–L4)	45.22 ± 4.96	43.42 ± 3.04	42.40 ± 2.66	0.33
Femoral neck	4.85 ± 0.35	4.91 ± 0.38	4.81 ± 0.24	0.13
Inter-trochanter	18.66 ± 2.76	16.38 ± 2.90	17.13 ± 1.76	0.38
Great trochanter	10.48 ± 1.60	9.75 ± 1.53	9.40 ± 0.86	0.34
Ward’s triangle	1.17 ± 0.09	1.16 ± 0.09	1.19 ± 0.09	0.14
*BMC (g)*				
Lumbar (L2–L4)	44.04 ± 9.69	43.78 ± 6.92*	38.95 ± 8.00	0.29
Femoral neck	3.74 ± 0.62	3.82 ± 0.73*	3.40 ± 0.66	0.27
Inter-trochanter	20.70 ± 4.68	18.00 ± 4.48	17.11 ± 2.58	0.39
Great trochanter	7.43 ± 1.90	6.87 ± 1.46	6.30 ± 1.01	0.32
Ward’s triangle	0.73 ± 0.18	0.75 ± 0.22	0.72 ± 0.25	0.06
*BMD (g/cm*^*2*^*)*				
Lumbar (L2–L4)	0.967 ± 0.127	1.010 ± 0.139*	0.915 ± 0.163	0.27
Femoral neck	0.773 ± 0.137	0.786 ± 0.139*	0.706 ± 0.123	0.26
Inter-trochanter	1.109 ± 0.174*	1.096 ± 0.152*	0.999 ± 0.113	0.34
Great trochanter	0.705 ± 0.107	0.707 ± 0.111	0.671 ± 0.092	0.17
Ward’s triangle	0.618 ± 0.136	0.646 ± 0.191	0.595 ± 0.180	0.12

OB, One-handed backhand; TB, two-handed backhand; SG, swimmers’ group

^*^Greater than SG,* p* < 0.05

### A comparison of the BA, BMC and BMD at distal radius between the dominant and non-dominant arm

In terms of BA, there were no differences between dominant and non-dominant arms across groups (Table 3). However, BMC values and BMD values at distal radius for the OB group were higher than for the SG group (*p* < 0.05). Finally, there was no difference between the TB group and the OB group and between the TB group and the SG group in terms of BMC and BMD.

**Table 3:** BA, BMC and BMD at distal radius in dominant and non-dominant arm (means ± SD)

	OB	TB	SG	Effect size (*f*)
*BA (cm*^*2*^*)*				
Dominant	21.57 ± 2.00	21.06 ± 3.08	20.78 ± 1.74	0.14
Non-dominant	21.08 ± 2.03	20.58 ± 2.54	20.29 ± 1.79	0.15
Δ_%_ (%)	2.54 ± 5.87	2.01 ± 4.62	2.49 ± 3.43	0.05
*BMC (g)*				
Dominant	10.86 ± 1.86	10.22 ± 2.10	9.97 ± 1.51	0.21
Non-dominant	9.85 ± 2.23	9.56 ± 1.89	9.69 ± 1.41	0.06
Δ_%_ (%)	12.39 ± 13.42*	7.07 ± 7.08	2.88 ± 3.51	0.49
*BMD (g/cm*^*2*^*)*				
Dominant	0.501 ± 0.045	0.488 ± 0.073	0.480 ± 0.062	0.14
Non-dominant	0.463 ± 0.072	0.465 ± 0.070	0.479 ± 0.062	0.10
Δ_%_ (%)	9.53 ± 10.74*	5.00 ± 5.61	0.40 ± 2.43	0.60

OB, One-handed backhand; TB, two-handed backhand; SG, swimmers’ group

^*^Greater than SG,* p* < 0.05

### Inter-arm asymmetry at the distal radius

**Fig. 1:**
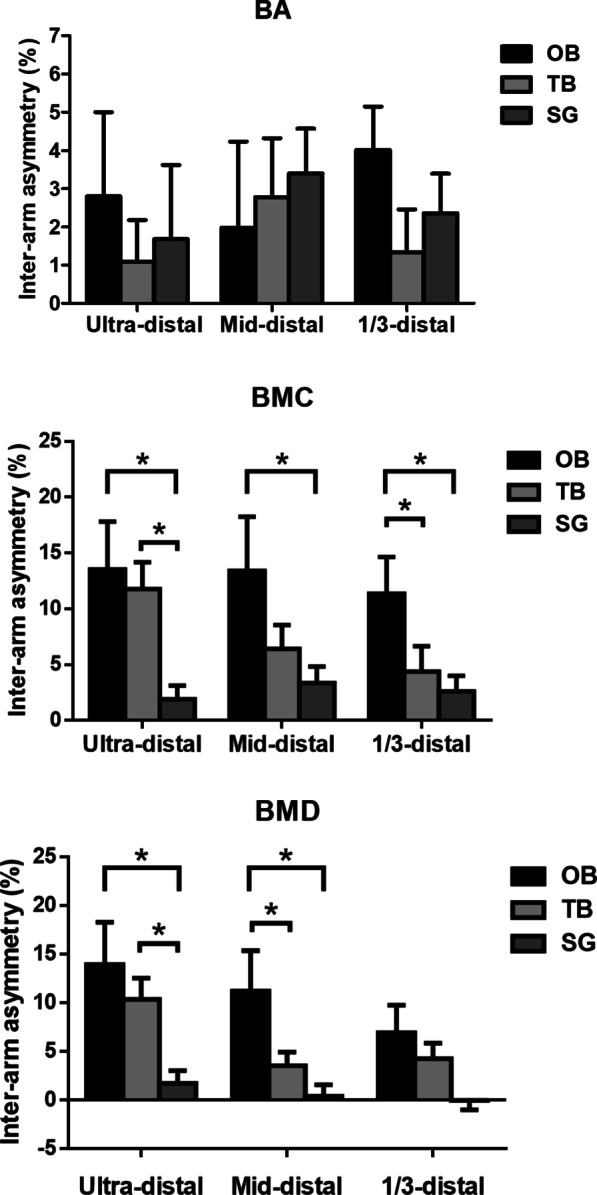
Inter-arm asymmetry of BA, BMC and BMD at three regions within distal radius (means ± SD) *Significantly different,* p* < 0.05. OB, one-handed backhand; TB, two-handed backhand; SG, swimmers’ group

The differences in inter-arm asymmetry (%) at the distal radius, including the UD, the MID and the 1/3 distal values are shown in Fig. 1. The BA values at the UD, the MID and the 1/3 distal were not significantly different between the groups. The OB group showed greater inter-arm BMC asymmetry across all three parts of the distal radius compared to the SG group (*p* < 0.05), while the OB group showed higher BMC asymmetry at 1/3 distal compared to the TB group. The BMC asymmetry of the TB group at the UD was greater than that of the SG group (*p* < 0.05). Inter-arm BMD asymmetry at UD, for both OB and TB groups, were higher than that shown by the SG group (*p* < 0.05). Finally, the OB group showed higher BMD asymmetry at MID than both the TB and SG groups (*p* < 0.05).

### Muscle strength in the dominant and non-dominant arms

Among the OB group, the grip strength of the dominant arm was higher than that of the SG group (*p* < 0.05, Table 4). When inter-arm asymmetry (%) was examined, the OB group showed greater than either the TB group or the SG group (*p* < 0.05). There was no difference in wrist flexion across the three groups that affected either the dominant asymmetry, the non-dominant arm asymmetry or the inter-arm asymmetry (%) (Table 4).

**Table 4:** Grip strength and isometric wrist flexion strength in dominant and non-dominant arm (means ± SD)

	OB	TB	SG	Effect size (*f*)
*Grip strength (kg)*				
Dominant	29.04 ± 2.44^a^	26.89 ± 3.58	25.15 ± 4.92	0.44
Non-dominant	24.22 ± 3.59	24.54 ± 3.57	22.98 ± 4.80	0.17
Δ_%_ (%)	21.56 ± 14.03^ab^	10.20 ± 10.60	10.36 ± 10.83	0.45
*Wrist flexion (Nm)*				
Dominant	9.29 ± 1.63	8.43 ± 1.28	8.66 ± 2.45	0.07
Non-dominant	8.51 ± 1.59	8.01 ± 1.41	8.55 ± 2.07	0.15
Δ_%_ (%)	10.72 ± 17.02	6.07 ± 11.52	1.06 ± 11.09	0.30

One-handed backhand, OB; two-handed backhand, TB; swimmers’ group, SG

^a^Greater than SG, ^b^Greater than TB;* p* < 0.05

## Discussion

In agreement with a previous study [22], we found that postmenopausal recreational tennis players showed greater BMC and BMD for the lumbar spine, femoral neck and inter-trochanter areas than swimmers. In the current study, most of participants had passed their peak bone mass age (i.e., ~ 35 years old) while starting playing tennis or swimming, suggesting that the recruited participants could represent the post-puberty group. Thus, our results have suggested that long-term tennis playing has positive effects on the bone tissue of postmenopausal women. The comparison of bone factors among three groups (OB, TB and SG) are discussed in the following section.

### The effect of different backhand stroke techniques on BMC and BMD values for the lumbar spine and left hip joint

Previous studies have only compared upper body bone tissue strength after tennis exercise [6]. This is the first study, to our best knowledge, to investigate whether the two different tennis backhand strokes techniques influence lower body bone tissue in different ways. We found that, for the TB group, both the BMC and the BMD values for the lumbar spine and femoral neck were significantly higher than for the SG group, which supports our hypothesis that performing two-handed backhand strokes increases both the core and lower body bone tissue health among postmenopausal women. Those differences were not observed between the OB group and the SG group. One possible explanation could be that two-handed backhand players might use larger joint movement by the rear leg, which could potentially result in a larger axial torque while rotating the hip and trunk [14, 15]. As a result, the two-handed backhand stroke might generate more mechanical loading on both the lumbar spine and the femoral neck. This would result in an increase in bone strength when this technique is used repeatedly over time. However, despite using a double-handed back-hand stroke might induce different mechanical loadings, compared with one-handed back-hand stroke, there were no differences between the TB and OB groups in BMC and BMD at the femoral neck and the BMD at the lumbar spine. The results suggest that the effects of this loading might not be big enough to affect core and lower body bone tissues. In addition, when the hip was examined, the BMD values at the inter-trochanter for both the TB and OB groups were higher than those of the SG group, which suggests that both backhand styles demonstrate the beneficial effects on BMD to the hip joint (e.g., inter-trochanter).

It has been shown previously that fractures of the lumbar spine make up about ~ 27% of all factures, and that hip and pelvis factures make up 14% and 7% of all factures, respectively [23]. Based on our findings, we propose that if the purpose of exercise among postmenopausal women is to maintain bone strength and preserve BMC and BMD then tennis as exercise would be a viable choice. More specifically, using a double-handed backhand would be more favorable regarding lumbar spine and femoral neck maintenance compared with swimming.

### The effect of different backhand strokes on the dominant and non-dominant arms

Stronger distal radius bone strength is likely to help prevent and/or reduce the risk of wrist fracture when a person falls [24]. In the current study, we did not find any difference in any of the assessed distal radius factors for either the dominant or non-dominant arm when swimmers were compared to all tennis players. One of the possible explanations is that water provides resistance to both hands during swimming, which might potentially help to maintain both BMC and BMD in the distal radius of swimmers. Studies have shown that the BMC and BA at upper extremities (e.g., distal radius and humeral shaft) are greater in swimmers than in non-exercise sedentary individuals [25, 26], suggesting that muscle induced mechanical loading/stimulation during swimming might help enhance bone strength (i.e., muscle-bone relationship) in the SG group in the current study. Therefore, despite swimming not being a weight-bearing loading impact exercise, we might not see an observable difference between the two groups. Accordingly, in tennis exercise regardless backhand styles and swimming exercise both could potentially reduce the risk of wrist fracture.

We hypothesize that the different forms of long-term tennis backhand play also might influence inter-arm asymmetry [10]. As anticipated, inter-arm asymmetry at distal radius for both BMC and BMD values among the OB group was greater than among the SG, but this was not true for the TB group. Our findings support previous results regarding unilateral exercise while using the one-handed backhand technique [13]. This previous study reported that the inter-arm difference for BMC among recreational postmenopausal tennis players was approximately 8% [6], which is close to our result for the TB group (7%) but lower than our result for the OB group (12%). However, which backhand technique was used by these players was not mentioned in their study, so we are unable to directly validate the 5% difference between the OB and TB groups. Studies have shown that there is a positive correlation between mechanical loading frequency and osteogenesis [27]; thus it would seem that mechanical stimulation is able to influence bone strength. In such circumstances, internal muscular forces are thought to be the greatest stressors on bones [28]. The non-dominant arm will receive more mechanical stimulation when performing two-handed backhand strokes among the TB group. This is accompanied by a greater inter-arm difference in arm muscle strength for the OB groups than the TB group in present study. Thus we presume that there may be a dose–response relationship occurring within the present study between the mechanical loadings that the bone receives and the enhancement in bone mass, which in turn leads to a diminished inter-arm asymmetry among the TB group.

### The effect of the two different backhand strokes on the distal radius

In our study, both the OB and TB groups showed greater inter-arm differences for BMC and BMD at the ultra-distal radius compared to SG. Furthermore, the BMC inter-arm difference for the OB group was greater than that for the TB and SG groups at 1/3 distal and this was also true for the BMD values at mid-distal radius. The ultra-distal region of the radius is essentially trabecular, whereas the mid-distal and 1/3 distal regions are mainly composed of cortical bone. Collectively, these results are in line with previous findings that cortical bone appears to play the main role in responding to tennis-induced mechanical loadings after adulthood or in older individuals who start playing [17, 29, 30]. In our study, this is manifested more among participants who habitually perform two-handed backhand strokes. This type of exercise seems to maintain the cortical bone within the distal radius of the non-dominant arm and thus potentially reduces the risk of fracture affecting this region.

### Considerations and limitations

There are some limitations affecting the present study. First, due to the nature of both tennis and swimming as forms of exercise in that, the former is intermittent and the latter is continuous, it is hard to precisely match the total amount of time spent exercising and this is reflected in our results. Furthermore, swimming is a non-weight bearing and non-impact type of exercise; therefore, it is possible within our study to discriminate between long-term participating in these two sports. Secondly, a non-exercise group was not recruited as part of the current study. However, based on our side-to-side comparisons, the confounding effects of genetic, nutritional and endocrine factors ought to have been eliminated, and the simple effects of the mechanical loadings applied to the bones are able to be observed via examination of the inter-arm (dominant *versus* non-dominant) differences. Therefore, the non-dominant arm of person using the one-handed backhand stroke can be considered to be the control in this part of the study. Thirdly, even though we have made sure that all participants were not receiving medication and supplements that might affect bone metabolism, we still cannot rule out the potential effects of different life styles and nutrition status on bone health [31]. Finally, the different ratios of top-spin *versus* back-spin (slice) might be used in the OB group and whether using different one-handed backhand technique could influence bone factors warrant future investigation.

## Conclusion

Long-term tennis exercise by postmenopausal women has positive benefits in terms of bone health compared to swimming exercise. More specifically, our findings suggest that using tennis backhand strokes in a two-handed manner regularly is able to decrease BMC and BMD inter-arm asymmetry for of the cortical bone at distal radius, as well as helping to maintain higher lumbar spine and hip BMC and BMD values. All of the above increases in bone health, when present in postmenopausal women, might potentially reduce the risk of fracture and osteoporosis in these specific regions.

## Data Availability

Not applicable.

## Abbreviations

ANCOVA: Analysis of covariance
ANOVA: Analysis of variance
BA: Bone area
BMC: Bone mineral content
BMD: Bone mineral density
DEXA: Dual-energy X-ray absorptiometry
MID: Mid-distal
OB: One-handed backhand
SD: Standard deviation
SG: Swimmers’ group
TB: Two-handed backhand
TMU: Taipei Medical University
UD: Ultra-distal
1/3: One-third radius
